# Differential Binding of Autoantibodies to MOG Isoforms in Inflammatory Demyelinating Diseases

**DOI:** 10.1212/NXI.0000000000001027

**Published:** 2021-06-15

**Authors:** Kathrin Schanda, Patrick Peschl, Magdalena Lerch, Barbara Seebacher, Swantje Mindorf, Nora Ritter, Monika Probst, Harald Hegen, Franziska Di Pauli, Eva-Maria Wendel, Christian Lechner, Matthias Baumann, Sara Mariotto, Sergio Ferrari, Albert Saiz, Michael Farrell, Maria Isabel S. Leite, Sarosh R. Irani, Jacqueline Palace, Andreas Lutterotti, Tania Kümpfel, Sandra Vukusic, Romain Marignier, Patrick Waters, Kevin Rostasy, Thomas Berger, Christian Probst, Romana Höftberger, Markus Reindl

**Affiliations:** From the Clinical Department of Neurology (K.S., P.P., M.L., B.S., H.H., F.D.P., M.R.), Medical University of Innsbruck, Austria; Euroimmun Medizinische Labordiagnostika AG (S. Mindorf, N.R., C.P.), Lübeck, Germany; Institute for Quality Assurance (ifQ) affiliated to Euroimmun (M.P.), Lübeck, Germany; Department of Pediatrics (E.-M.W.), Olgahospital/Klinikum Stuttgart, Germany; Department of Pediatrics I (C.L., M.B.), Medical University of Innsbruck, Austria; Neurology Unit (S. Mariotto, S.F.), Department of Neuroscience, Biomedicine, and Movement Sciences, University of Verona, Italy; Neuroimmunology and Multiple Sclerosis Unit (A.S.), Service of Neurology, Hospital Clinic, Institut d'Investigacions Biomèdiques August Pi i Sunyer (IDIBAPS), Universitat de Barcelona, Spain; Beaumont Hospital (M.F.), Dublin, Ireland; Oxford Autoimmune Neurology Group (M.I.S.L., S.R.I., J.P., P.W.), Nuffield Department of Clinical Neurosciences, University of Oxford, UK; Neuroimmunology and MS Research (A.L.), Department of Neurology, University Hospital Zurich & University of Zurich, Switzerland; Institute of Clinical Neuroimmunology (T.K.), Biomedical Center and University Hospital, Ludwig-Maximilians University, Munich, Germany; Department of Neurology (S.V., R.M.), Hospices civils de Lyon, Hôpital neurologique Pierre Wertheimer, France; Paediatric Neurology (K.R.), Witten/Herdecke University, Children's Hospital Datteln, Germany; Department of Neurology (T.B.), Medical University of Vienna, Austria; and Division of Neuropathology and Neurochemistry (R.H.), Department of Neurology, Medical University of Vienna, Austria.

## Abstract

**Objective:**

To analyze serum immunoglobulin G (IgG) antibodies to major isoforms of myelin oligodendrocyte glycoprotein (MOG-alpha 1-3 and beta 1-3) in patients with inflammatory demyelinating diseases.

**Methods:**

Retrospective case-control study using 378 serum samples from patients with multiple sclerosis (MS), patients with non-MS demyelinating disease, and healthy controls with MOG alpha-1-IgG positive (n = 202) or negative serostatus (n = 176). Samples were analyzed for their reactivity to human, mouse, and rat MOG isoforms with and without mutations in the extracellular MOG Ig domain (MOG-ecIgD), soluble MOG-ecIgD, and myelin from multiple species using live cell-based, tissue immunofluorescence assays and ELISA.

**Results:**

The strongest IgG reactivities were directed against the longest MOG isoforms alpha-1 (the currently used standard test for MOG-IgG) and beta-1, whereas the other isoforms were less frequently recognized. Using principal component analysis, we identified 3 different binding patterns associated with non-MS disease: (1) isolated reactivity to MOG-alpha-1/beta-1 (n = 73), (2) binding to MOG-alpha-1/beta-1 and at least one other alpha, but no beta isoform (n = 64), and (3) reactivity to all 6 MOG isoforms (n = 65). The remaining samples were negative (n = 176) for MOG-IgG. These MOG isoform binding patterns were associated with a non-MS demyelinating disease, but there were no differences in clinical phenotypes or disease course. The 3 MOG isoform patterns had distinct immunologic characteristics such as differential binding to soluble MOG-ecIgD, sensitivity to MOG mutations, and binding to human MOG in ELISA.

**Conclusions:**

The novel finding of differential MOG isoform binding patterns could inform future studies on the refinement of MOG-IgG assays and the pathophysiologic role of MOG-IgG.

Serum immunoglobulin G (IgG) autoantibodies against myelin oligodendrocyte glycoprotein (MOG-IgG) are associated with a spectrum of neurologic diseases including optic neuritis (ON), acute disseminated encephalomyelitis (ADEM), myelitis, seizures, encephalitis, and with brainstem and/or cerebellar involvement.^1-9^ In addition, MOG-IgG appears to be supportive to discriminate these disorders from multiple sclerosis (MS)^10,11^ as reflected by the first diagnostic recommendations for MOG-IgG–associated disorders (MOGADs).^1,6,12^ Furthermore, MOGADs are not only characterized by clinical but also neuropathologic features.^13,14^

Although most studies use live cell-based assays (CBAs) with the MOG alpha (α) 1 isoform for the measurement of MOG-IgG, previous results were often discrepant, because of different MOG expression vectors (full-length vs extracellular domain), cell lines, read-out systems (immunofluorescence [IF] vs flow cytometry), and other test variations, which aimed to increase specificity and eliminate nonspecific low-titer positivity.^1,15-17^ Several studies attempted to define the molecular epitopes of MOG-IgG with the help of amino acid substitutions or deletions and discovered distinct binding patterns.^18-21^ The most frequent epitopes were located in the loops between the β sheets of the extracellular Ig domain of human MOG (MOG-ecIgD). These findings were extended by other studies showing that only a subset of human MOG-IgG is also reactive to rodent MOG epitopes^18,19,22,23^ and pathogenic in vitro or in vivo.^19,23,24^

Earlier studies using MOG-ecIgD as an antigen for immunoassays have indicated a lower sensitivity compared with full-length MOG (with the α1 isoform as the consensus sequence).^10,25^ Although these results indicated binding differences to different MOG variants, no study so far has analyzed antibody responses to different MOG isoforms. Like most other human myelin genes, the MOG gene undergoes extensive alternative splicing and multiple different MOG isoforms have been described in primates, but not in rodents.^26-29^ Although the extracellular Ig domain is present in all these isoforms, they show profound differences in the composition of the intracellular C terminus resulting in alpha (α) or beta (β) isoforms.

The aim of our study was to analyze the serum IgG antibody response to additional MOG isoforms (α2, α3, β1, β2, and β3; figure 1) in MOGα1-seropositive and MOGα1-seronegative patients and controls. Furthermore, we analyzed whether the use of additional MOG isoforms (either alone or in combinations) improves the specificity of MOG-IgG CBAs.

**Figure 1:**
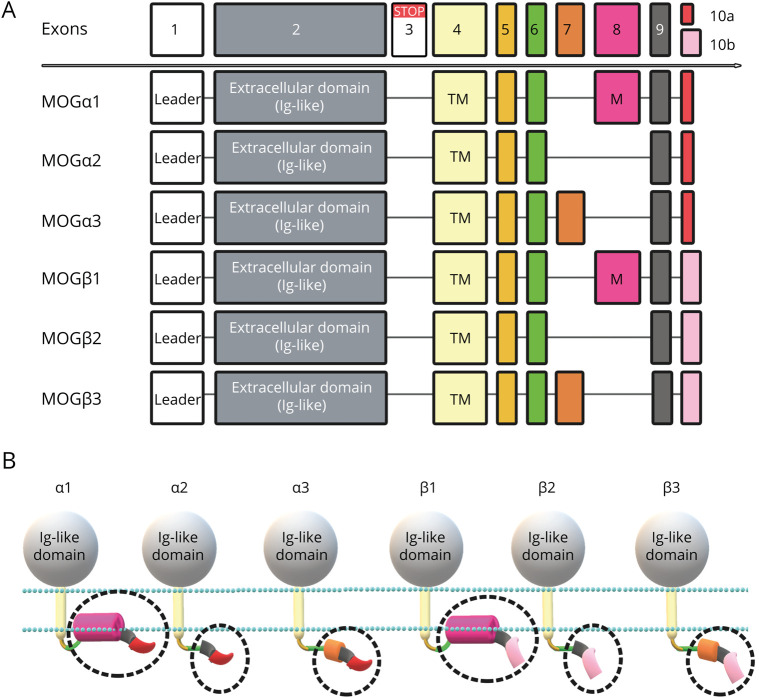
Splicing Variants and Protein Isoforms of Human MOG α1-3 and β1-3 (A) Exon composition of different MOG precursor transcript variants: leader = signal peptide, removed during maturation, exon 3 = contains a stop codon and is present in transcripts for soluble MOG (not depicted in this schematic), TM = encodes the single transmembrane domain, M = encodes the membrane-associated intracellular part of transcripts α1 and β1, exon 7 = specific for transcripts α3 and β3. The last 3′ exons encode specific sequences for MOGα1-3 (10a) and MOGβ1-3 (10b). (B) Protein isoforms of human MOG α1-3 and β1-3. The extracellular Ig-like domain is present in all isoforms (gray), whereas the specific intracellular composition is shown slightly enlarged within the dashed circles using colors fitting to the coding exons described above. MOG = myelin oligodendrocyte glycoprotein.

## Methods

### Patients and Controls

We performed a retrospective case-control study including serum samples from 378 patients with inflammatory demyelinating diseases and healthy controls (HCs) with known MOG-IgG serostatus recruited from the participating centers for this study (Innsbruck and Vienna, Austria; Verona, Italy; Barcelona, Spain; Dublin, Ireland; Oxford, UK; Zurich, Switzerland; Munich and Lübeck, Germany; and Lyon, France).^2,10,16,22^ We included samples from children (age <18 years) and adults (≥18 years) with an aquaporin-4–seronegative non-MS demyelinating disease typically associated with MOG-IgG (n = 214, thereof 191 seropositive MOGAD), MS (n = 64),^11^ and HCs (n = 100). We also included MOG-IgG–positive MS (n = 8) and HC (n = 3) samples collected at different centers, and therefore, MOG-IgG seropositivity in MS and HCs is overrepresented compared with previous studies. Samples were sent to Innsbruck and reclassified there as MOG-IgG negative (n = 176; 47%; MOG-IgG(H + L) titer 0 to 1:80; MOG-IgG(Fc) negative) or MOG-IgG positive (n = 202; 53%; MOG-IgG(H + L) titer 1:160 to 1:20,480; MOG-IgG(Fc) positive) using a combination of 2 different live IF MOGα1 CBAs (CBA-IF) as recently described.^16^ In addition, 106 follow-up samples of 35 individuals were analyzed with a median observation period of 4.1 years (range 0.8–12.1 years). Because a detailed clinical and radiologic description was not the primary scope of this study, we summarized clinical data of non-MS patients in predominant clinical phenotypes: opticospinal (isolated optic neuritis, myelitis, brainstem syndrome, or a combination of those, a proportion of these patients fulfilled the current diagnostic criteria for neuromyelitis optica spectrum disorder^30^), cerebral (ADEM according to current diagnostic criteria,^31^ multiphasic disseminated encephalomyelitis, brainstem encephalitis, or encephalitis), or a combination of both (ADEM followed by optic neuritis and opticospinal with cerebral symptoms) and disease courses (monophasic vs recurrent). Clinical and demographic data according to the MOG-IgG serostatus are shown in table e-1, links.lww.com/NXI/A498.

Neuropathologic investigations were available from 6 MOG-IgG–positive cases: 2 with biopsy and autopsy, 3 with biopsy, and 1 with autopsy. Four of these patients had previously been described as case reports and included in a series of patients to describe the pathology of CNS demyelination accompanied by MOG-IgG.^13^ Serum/CSF samples and biopsy/autopsy specimens were referred to the Division of Neuropathology and Neurochemistry, Department of Neurology, Medical University of Vienna for diagnostic purposes and stored at the institutional biobank.

### Standard Protocol Approvals, Registrations, and Patient Consents

This study was approved by the ethical committees of the Medical University of Innsbruck, Austria (AM3041A and AM4059), Medical University of Vienna (EK 1636/2019 and 1123/2015), University of Oxford, UK (REC 16/SC/0224), Hospital Clinic of Barcelona, Spain (2010/5680), and University of Zürich, Switzerland (KEK ZH 2013-0001). Twenty serum samples were obtained from the NeuroBioTec—Hospices Civils de Lyon biobank, 10 samples from the Neuropathology-Verona biobank, and 10 samples from the Biobank of LMU Munich, Germany (Biobank 163-16). All patients or their caregivers and controls gave written informed consent. All samples from participating centers were anonymized before sending them to Innsbruck, Austria.

### Cloning of MOG Isoform Constructs

The following human MOG isoforms shown in figure 1 were expressed as C-terminal enhanced green fluorescent protein fusion proteins (EGFP) in this study: MOGα1 (NCBI CCDS34370.1; UniProt Q16653-1), MOGα2 (NCBI CCDS47394.1; UniProt Q16653-2), MOGα3 (NCBI CCDS34369.1; UniProt Q16653-3), MOGβ1 (NCBI CCDS4667.1; UniProt Q16653-5), MOGβ2 (NCBI CCDS34367.1; UniProt Q16653-6), and MOGβ3 (NCBI CCDS34366.a; UniProt Q16653-7). The detailed cloning strategies are described in Supplementary methods. In addition, we used pEGFP-N1 (Takara Clontech) expression vectors encoding mouse MOG, rat MOG, and the human MOGα1 mutants N31Q (N-glycosylation site^18,20,21,23,32^), P42S (mouse/rat specific, immunodominant human MOG epitope^18,21,23,32^), E64K (possible binding motif for complement C1q^32^), A75S (rat specific^18,32^), R86Q (mouse/rat specific^18,32^), and H103A + S104E (epitope of the mouse monoclonal MOG antibody 8-18-C5, important human MOG epitope^18,21,23,32,33^) as described in Supplementary methods.

### Immunoassays for the Detection of MOG-IgG Binding to Different MOG Isoforms

All live CBAs were performed using HEK293 cells transfected with the individual expression vectors encoding the different MOG isoforms described above. Because of the amount of analyses per sample, evaluation of antibody binding to the different isoforms and animal species was performed on the same day to reduce the number of freeze-thawing cycles. All CBAs (live CBA-IF, live CBA–fluorescence-activated cell sorting [FACS], and a commercial fixed CBA-IF from Euroimmun) and ELISAs were performed according to recently published detailed protocols.^16^ We used a combination of 2 assays with secondary antibodies to human IgG(H + L) and confirmation by secondary antibodies to human IgG(Fc) to exclude isolated IgM reactivity. Final results were given in titer level (CBA-IF) or binding ratio (CBA-FACS). The binding of serum samples to human, mouse, and rat myelin was analyzed by a tissue-based IF assay as described before.^19^

### Competitive Binding Assay Using the Soluble Extracellular Domain of MOG

We performed competitive binding assays by CBA-FACS^16^ to investigate whether MOG-IgG recognizes soluble human MOG-ecIgD and to determine antibody affinities. Serum samples were diluted according to their FACS median binding ratio to standardize MOG-IgG amounts. Selection of samples was based on the MOG isoform binding pattern observed in live CBA-IF. Diluted serum samples were incubated with increasing amounts (0, 1.6, 3.2, and 9.6 μM) of soluble human MOG-ecIgD (provided by Euroimmun, Lübeck)^16^ for 1 hour at room temperature and spun down at 10,000*g* for 5 minutes. After this preabsorption step, samples were analyzed by CBA-FACS. Nonlinear regression analysis was used to analyze competitive binding and calculate IC50 values.

### Statistical Analysis

The primary hypothesis of this study was that there are differential disease-specific IgG antibody responses to MOG isoforms (α1, α2, α3, β1, β2, and β3). This hypothesis was tested for antibody titers using the Friedman nonparametric test, Spearman's rank nonparametric correlational analyses, and receiver operating curve analysis. Principal component analysis (PCA) was used to classify groups of log2-transformed MOG-IgG titers. This unsupervised, unbiased multivariate analysis approach was used to identify the set of variables (MOG isoforms) accounting for the greatest variation present in the data set. Loading plots were generated to visualize the combination of MOG isoforms responsible for clustering. The analyses of all secondary and other endpoints focused on estimates (common OR; positive and negative predictive values [PPV and NPV]; percentages, medians, and median differences) and 95% CI or 25th to 75th interquartile ranges. Groups were compared using Kruskal-Wallis or χ^2^ tests. Statistical significance was defined as 2-sided *p* value < 0.05 (adjusted for multiple comparisons using Bonferroni's correction). Statistical analyses were performed using IBM SPSS software (IBM SPSS Statistics; Version 26.0. Armonk, NY: IBM Corp.) or GraphPad Prism 9 (GraphPad Software, La Jolla, CA).

### Data Availability

The data set used and analyzed during the current study is included in the main text or the supplementary files or is available from the corresponding author on reasonable request. Expression plasmids for the different isoforms will be deposited with Addgene.

## Results

### Differential Antibody Binding to MOG Isoforms α1, α2, α3, β1, β2, and β3

**Figure 2:**
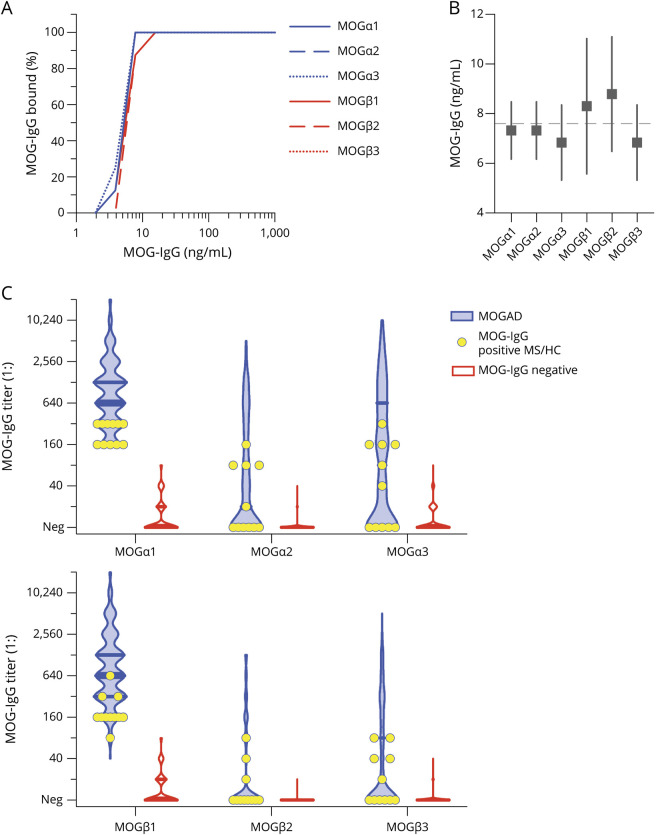
Differential Binding of Human MOG-IgG to MOG Isoforms α1, α2, α3, β1, β2, and β3 (A) Serial dilutions of humanized monoclonal mouse MOG antibody 8-18-C5. The graph shows the percentage of 8 replicates determined MOG-IgG positive in live CBA-IF transfected with individual MOG isoforms. (B) Mean sensitivity (with 95%) for MOG-IgG in live CBA-IF using the different MOG isoforms as antigens. The dashed line indicates the overall mean sensitivity. (C) Quantitative binding of MOG-IgG to MOG isoforms α1-3 (upper panel) and β1-3 (lower panel) for all 378 samples according to their MOG-IgG serostatus (202 positive and 176 negative). Seropositive samples with an aquaporin-4–seronegative non-MS demyelinating disease typically associated with MOG-IgG (MOGAD, n = 191) are shown as blue violin plots, and seropositive samples from patients with MS (n = 8) or healthy controls (n = 3) are shown as yellow circles. Seronegative samples are shown as red violin plots. Medians and interquartile ranges are indicated by the bars within violin plots. CBA = cell-based assay; HCs = healthy controls; IF = immunofluorescence; MOG = myelin oligodendrocyte glycoprotein; MOG-ecIgD = extracellular MOG immunoglobulin.

Because previous studies could not consistently clarify which MOG epitopes are immunodominant, we used a different approach and compared the binding of human MOG-IgG with MOG isoforms α1, α2, α3, β1, β2, and β3. We analyzed serum samples from 378 individuals with MOG-IgG serostatus determined using MOGα1 live CBA (176 negative and 202 positive) and single transfections of HEK293 cells with the other MOG isoforms. Cell surface expression of isoforms was demonstrated by performing live CBA with serial dilutions of the humanized mouse monoclonal MOG antibody 8-18-C5 showing comparable detection endpoints (figure 2, A and B). Despite small differences in transfection efficiencies, MOG isoforms showed comparable specific surface binding of humanized MOG antibody 8-18-C5 (figures e-2 and e-3, links.lww.com/NXI/A498). Therefore, all extracellular domains of the different MOG isoforms were determined equally available for antibody binding.

Overall, the strongest antibody reactivities were directed against the longest MOG isoforms α1 (median titer 1:160, 25th–75th percentile 0–640) and β1 (1:160, 0–640), whereas isoforms α2 (0, 0–20), α3 (0, 0–80), β2 (0, 0–0), and β3 (0, 0–0) were less frequently recognized (*p* < 0.001). There were strong correlations between α1 and β1 (Spearman's ρ 0.990) and between α2, α3, β2, and β3 (Spearman's ρ 0.677–0.845), whereas the correlations between α1/β1 and α2/α3/β2/β3 were only moderate (Spearman's ρ 0.427–0.545).

In a next step, we analyzed whether the individual MOG isoforms are useful for discriminating a non-MS demyelinating disease from MS or HC. As can be seen in figure 2C, low-titer antibodies against all MOG isoforms were also present in a few cases with MS and in HCs. The positive and negative predictive values for all MOG isoforms according to titer steps are shown in table e-3, links.lww.com/NXI/A498. The cutoff values associated with a PPV (for MOG-IgG associated with a clinical MOGAD phenotype) of ∼95% were ≥1:160 for MOG α1 (190/214 non-MS vs 11/164 controls), α3 (89/214 non-MS vs 4/164 controls), and β1 (185/214 non-MS vs 10/164 controls), ≥1:40 for MOG α2 (86/214 non-MS vs 5/164 controls) and β2 (52/214 non-MS vs 2/164 controls), and ≥1:80 for MOG β3 (58/214 non-MS vs 2/164 controls).

### Identification of Distinct Non-MS–Associated MOG Isoform Binding Patterns

**Figure 3:**
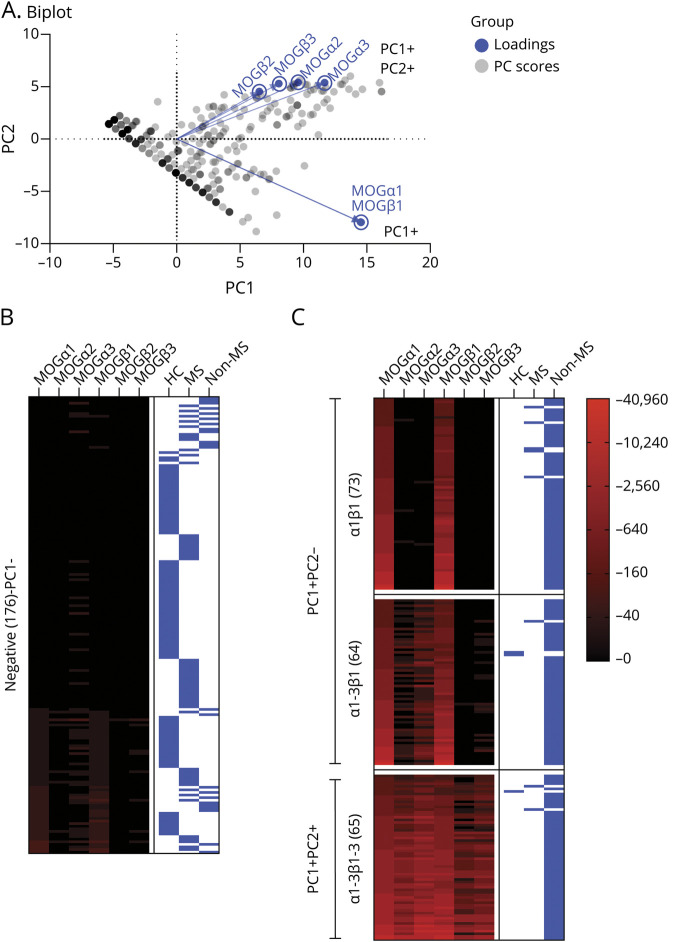
Binding Patterns of Human MOG-IgG to MOG Isoforms α1, α2, α3, β1, β2, and β3 (A) Scatter dot plot showing the principal component (PC) scores of all samples and the loading scores of all MOG isoforms to visualize parameters responsible for clustering. This identified different binding pattern clusters associated with PC1 (α1 and β1) and/or PC2 (α2, α3, β2, and β3). Heatmap of the quantitative results (MOG-IgG titers) for all MOG-IgG–seronegative samples (B) or MOG-IgG–seropositive samples (C) according to their MOG isoform binding pattern (negative, α1β1, α1-3β1, and α1-3β1-3). Each column is an individual MOG isoform, and each row is an individual serum sample with MOG isoform binding patterns indicated on the left. MOG-IgG reactivities (titer 1:) are shown in different color intensities (legend with the log scale) and clinical diagnosis of non-MS, MS, and HC. (B) Individual MOG isoform IgG titers (median with the interquartile range) according to the identified binding patterns. The cutoff value for MOG-IgG positivity (α1 1:160) is indicated by the dashed blue line. (C) Percentage of patients with clinical diagnosis of non-MS (MOGAD), MS, and HC according to MOG isoform binding patterns. HCs = healthy controls; MOG = myelin oligodendrocyte glycoprotein; MOGAD = MOG-IgG–associated disorders; MOG-IgG = serum IgG antibodies against MOG.

**Figure 4:**
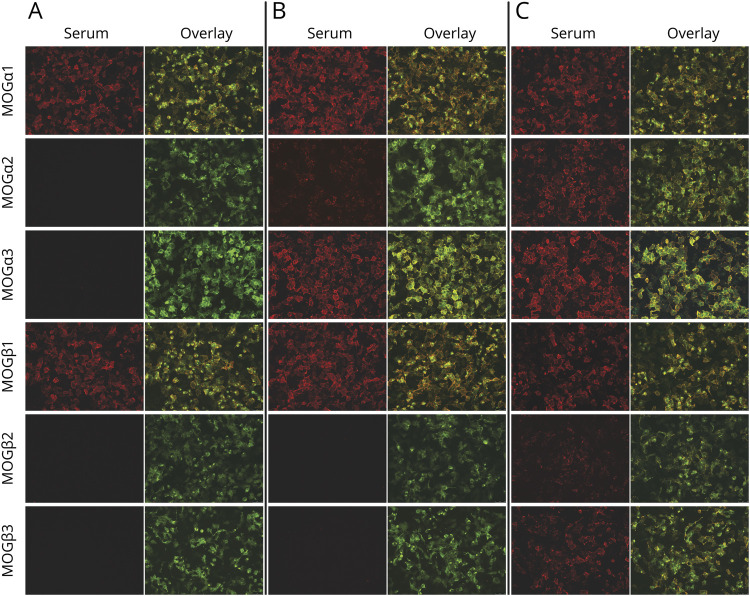
Representative MOG Isoform CBA-IF Stainings Representative live CBA-IF serum stainings for the 3 identified MOG isoform antibody binding patterns (A, α1β1; B, α1-3β1; C, α1-3β1-3). Only specific antibody (red) and overlay images (MOG-transfected cells are shown in green) were used to reduce image size (20× magnification). CBA = cell-based assay; IF = immunofluorescence; MOG = myelin oligodendrocyte glycoprotein.

In a next step, we investigated combinations of MOG isoforms using unsupervised PCA to generate a PCA scores plot of all samples and isoforms (figure 3A). The variables driving clustering are clearly differentiated in PC1 (all isoforms with strongest contributions of α1 and β1; 75.3% of variance, Eigenvalue 28.7) and PC2 (α2, α3, β2, and β3; 21.2% of variance, Eigenvalue 8.1) together explaining 96.5% of the total variance in the data set. The biplot shown in figure 3A indicates the following major groups: negative for PC1 (seronegative for MOG-IgG), positive for PC1 but negative for PC2, and positive for both PC1 and PC2. This separation into different binding clusters is shown in figure 3 and table 1. The “negative” cluster (PC1 negative, n = 176) was associated with MOG-IgG–negative (MOGα1 titer 0-1:80) non-MS, MS, and HC samples. By contrast, the other binding patterns were predominantly associated with non-MS and dominated by either PC1 or PC2: (1) equal binding to α1 and β1 and no binding to α2, α3, β2, and β3 (pattern α1β1, n = 73), (2) equal binding to α1 and β1, weaker binding to α2 and α3, and no binding to β2 and β3 (pattern α1-3β1, n = 64), and (3) binding to all MOG isoforms (pattern α1-3β1-3, n = 65). Three representative CBA-IF stainings for samples assigned to these isoform binding patterns are shown in figure 4.

**Table 1:**
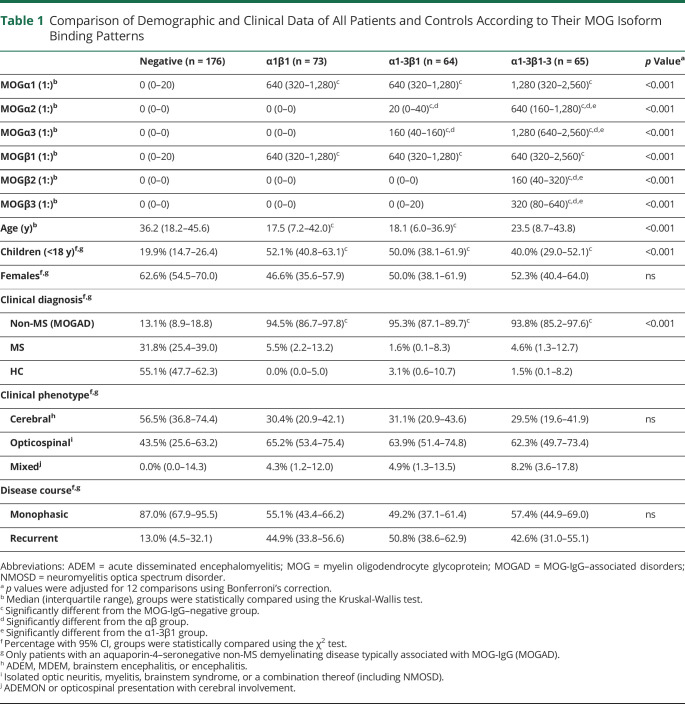
Comparison of Demographic and Clinical Data of All Patients and Controls According to Their MOG Isoform Binding Patterns

It is important that these binding patterns were associated with a non-MS demyelinating disease consistent with MOGAD when compared with the MOG-IgG–negative patients and controls (table 1): pattern α1β1 (PPV 94.5%, 95% CI 86.7 to 97.9), pattern α1-3β1 (PPV 95.3%, 95.3% CI 87.1 to 98.7), and pattern α1-3β1-3 (PPV 93.8%, 93.9% CI 85.2 to 97.6). The proportion of cases with MOGα1 titers ≥1:640 (high-titer MOG-IgG positive) specifically associated with MOGAD^16^ was higher in the α1-3β1-3 (74%) than in the α1-3β1 (58%) or α1β1 (59%) binding patterns.

Furthermore, we analyzed 106 available follow-up samples of 35 cases (7 α1β1, 15 α1-3β1, and 13 α1-3β1-3) to assess the temporal stability of these binding patterns. As shown in figure e-4, links.lww.com/NXI/A498, 3 of the 7 (43%) patients with α1β1, 10 of the 15 (71%) patients with α1-3β1, and 9 of the 13 (69%) patients with α1-3β1-3 binding patterns kept their isoform binding pattern. The other patients converted to α1β1 (3) or α1-3β1 (3), or became seronegative (7). There was no conversion from α1β1 to α1-3β1-3 or vice versa.

### Associations of Different MOG-IgG Binding Patterns With Non-MS Clinical Phenotype, Disease Course, and Neuropathology

Children with a non-MS disease course were more frequently associated with MOG isoform binding patterns α1β1 and α1-3β1, whereas adults were more frequently associated with α1-3β1-3 or seronegative MOG-IgG (table 1). The percentage of females was comparable between binding patterns. Furthermore, the percentage of patients with different clinical presentations of a non-MS demyelinating disease (cerebral, opticospinal, or mixed presentation) or monophasic or recurrent course at the last follow-up was comparable in all 3 MOG isoform binding patterns. Moreover, there was no association of IgG titers or seropositivity against the individual MOG isoforms with demographic or clinical parameters (tables e-4 and e-5, links.lww.com/NXI/A498).

Finally, we analyzed the neuropathologic features at disease/relapse onset and follow-up in the 6 cases with available biopsies and/or autopsies (figure e-5, links.lww.com/NXI/A498). One of these cases (perivenous demyelination) had α1β1 antibodies, 1 had the α1-3β1 binding pattern (perivenous demyelination, conversion to low-titer MOG-IgG at follow-up), and 4 had the α1-3β1-3 binding pattern (1 with perivenous and 3 with confluent demyelination, one of them with intrathecal MOG-IgG).

### MOG-IgG Binding Patterns to Different Isoforms Are Associated With a Differential Recognition of MOG Epitopes

**Figure 5:**
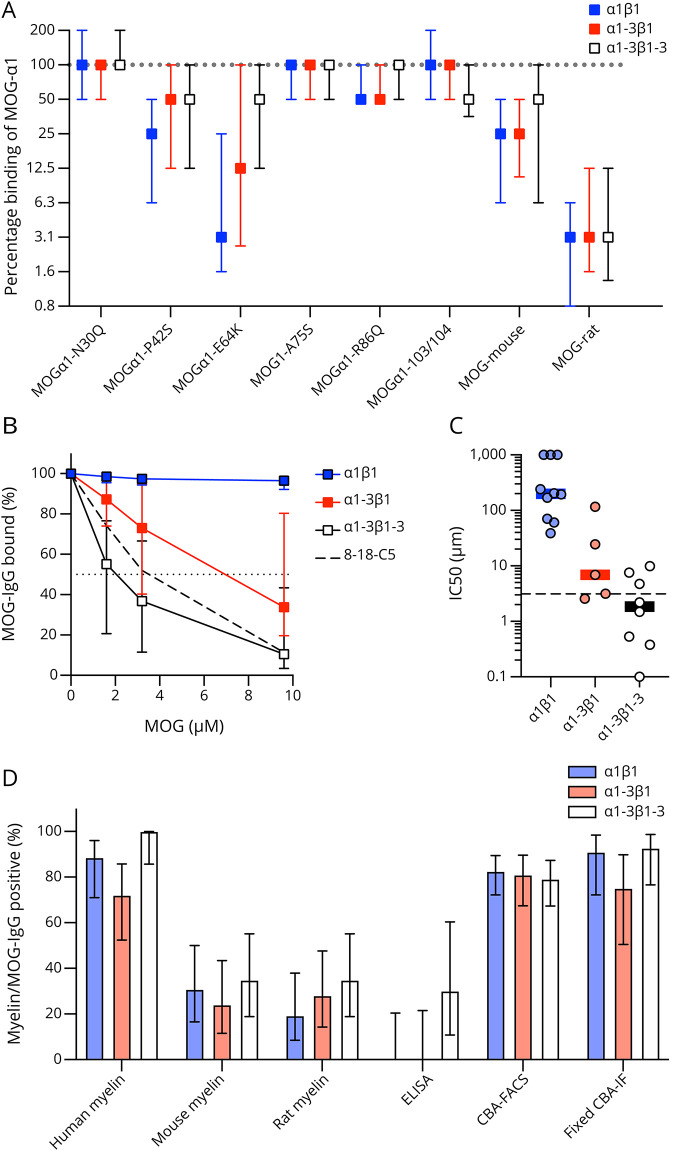
MOG-IgG Binding Patterns to Different Isoforms are Associated With a Differential Recognition of MOG Epitopes (A) Binding of MOG-IgG to the human MOGα1 mutants N31Q, P42S, E64K, A75S, R86Q, and H103A + S104E (103/104) and to mouse and rat MOG according to their MOG isoform binding patterns (α1β1, n = 64; α1-3β1, n = 43; α1-3β1-3, n = 46). The median differences compared to wild-type MOGα1 are shown as symbols with interquartile ranges (error bars). (B) Competition of binding to MOGα1 in a CBA-FACS assay by soluble MOG-ecIgD added in increasing concentrations (0, 1.6, 3.2, and 9.6 μM) for the 3 MOG isoform binding patterns (α1β1, n = 10; α1-3β1, n = 5; α1-3β1-3, n = 8) and for monoclonal antibody 8-18-C5 (dashed line). Squares indicate the median percentage bound, error bars indicate the interquartile ranges, and the value used for the calculation of IC_50_ (50% binding) is indicated by the dotted line. Groups were statistically compared using repeated measures 2-way analysis of variance. (C) IC_50_ values for the competition of binding to MOGα1 in a CBA-FACS assay by soluble MOG-ecIgD according to MOG isoform binding patterns (α1β1, n = 10; α1-3β1, n = 5; α1-3β1-3, n = 8). Individual data points are shown by scatter dots, and medians and interquartile ranges are indicated by lines and error bars. The IC_50_ value for monoclonal antibody 8-18-C5 is indicated by the dashed line. (D) Binding of human MOG-IgG according to the 3 MOG isoform binding patterns to human, mouse, and rat myelin in brain sections, as well as in ELISA (human MOG Ig domain), CBA-FACS (human MOGα1), and a commercial fixed CBA-IF (human MOGX11). The percentages of myelin/MOG-IgG–positive samples are shown as bars with 95% CI (error bars). CBA = cell-based assay; FACS = fluorescence-activated cell sorting; IF = immunofluorescence; MOG = myelin oligodendrocyte glycoprotein; MOGAD = MOG-IgG–associated disorders; MOG-ecIgD = extracellular MOG immunoglobulin domain; MOG-IgG = serum IgG antibodies against MOG.

We performed a number of additional experiments (reactivity to several mutations in the extracellular Ig domain of MOGα1, competitive binding experiments with soluble MOG-ecIgD, reactivity in CBA-FACS and ELISA, reactivity to fixed MOG expressing cells, and reactivity to human, mouse, and rat MOG in brain tissue) to elucidate the molecular mechanisms underlying the 3 different MOG isoform binding patterns in a subset of samples. Figure 5A shows the differential binding to human MOGα1 mutants, as well as mouse and rat MOG observed in the 3 MOG-IgG binding patterns in relation to MOGα1. The P42S and E64K mutations strongly affected MOG-IgG binding in all 3 binding patterns, with the strongest reduction seen in the α1β1 pattern. The N31Q, A75S, R86Q, and H103A + S104E mutations had comparable effects in all binding patterns. Binding to mouse and rat MOG was reduced in all 3 isoform binding patterns.

Next, we analyzed whether the binding to different MOG isoforms can be inhibited by competition with increasing concentrations (0, 1.6, 3.2, and 9.6 μM) of soluble human MOG-ecIgD in a CBA-FACS competitive binding assay. As shown in figure 5B, addition of soluble MOG-ecIgD was able to reduce the binding to MOGα1 in the α1-3β1-3 pattern comparable with the monoclonal MOG antibody 8-18-C5, but not in pattern α1β1. Furthermore, pattern α1-3β1-3 also had similar IC50 and affinities (0–10 µM, figure 5C). By contrast, antibodies of pattern α1β1 did not seem to bind to the soluble MOG-ecIgD. Of interest, pattern α1-3β1 appears to hold a position between the 2. Because median MOG-IgG titers were comparable (1:640 for all 3 binding patterns), we can exclude an antibody concentration effect. Similar findings were obtained using an MOG-IgG ELISA in which α1-3β1-3 antibodies showed better binding than the other patterns (figure 5D). By contrast, we observed no differences for reactivity in CBA-FACS, fixed CBA-IF, and antibody binding to human, mouse, or rat myelin.

## Discussion

Our results show that the strongest MOG-IgG responses are directed against the longest MOG isoforms α1 and β1, whereas the shorter isoforms α2, α3, β2, and β3 are less frequently recognized. Using PCA, we could confirm 3 distinct MOG isoform binding patterns specific for patients with MOG-IgG–associated non-MS demyelinating disease (MOGAD): (1) isolated binding to MOG α1 and β1 only (α1β1), (2) a mixed binding pattern with dominant recognition of α1 and β1 and at least 1 additional alpha MOG isoform (α1-3β1), and (3) binding to all MOG isoforms (α1-3 β1-3). Recently, we demonstrated that high-titer positive MOG-IgG (MOGα1 ≥1:640) are consistently positive in all live CBAs and specific for non-MS demyelinating disease, whereas low-titer positive MOG-IgG (MOGα1 1:160–1:320) are more frequently discordant and sometimes also found in patients with MS and HCs.^16^ This observation is also seen in our study with MOG-IgG–positive MS and HC cases found only in the low-titer positive group present in all 3 binding patterns. However, MOG-IgG seropositivity in MS and HCs in our study was overrepresented compared with previous studies.^1^

The recognition of different MOG isoforms by MOG-IgG is not associated with relevant differences in clinical presentations, disease course, or neuropathology. Because our study has several limitations such as its retrospective design, the heterogenous observation period, and missing information on treatment, this needs further investigation in a prospective study. However, demographic and clinical characteristics of our study population are consistent with previous reports.^1,2,5,6,9,12,22^ Our results indicate that α1β1 and α1-3β1 MOG-IgG could be more common in children and α1-3β1-3 MOG-IgG in adults. This observation is consistent with previous findings showing a dominant expression of the isoforms MOG α1 and β1 in the human brain, and especially in early development, only these 2 isoforms are detected.^28,29^

We used a number of additional immunologic investigations to clarify our findings. Only MOG-IgG of the α1-3β1-3, but not the α1β1, binding pattern also bound to soluble MOG-ecIgD with high affinity (20 μM–10 nM), which might explain why immunoprecipitation assays found MOG-IgG less frequently than CBAs^25,34^ and why attempts to isolate human MOG-IgG using affinity binding to MOG-ecIgD were only partly successful.^21,23^ This result is surprising because all MOG-IgG presumably bind to epitopes in the extracellular Ig domain, and therefore, they should compete similarly with MOG-ecIgG. Additional studies are now needed to elucidate the underlying molecular mechanisms of this finding. Furthermore, the fact that only a subset of human MOG-IgG (those of MOG α1-3β1-3 binding pattern) was reactive in MOG ELISA may explain discrepancies found in the literature.^16,21,25,35^ Finally, all 3 MOG isoform binding patterns show different sensitivities to mutations in the extracellular MOG Ig domain: whereas all patterns were sensitive to the P42S mutation associated with an immunodominant MOG epitope^18,21,23,32^ and the E64K mutation in the C″D loop,^36-38^ this effect was even more pronounced in the α1β1 binding pattern. Because the α1-3β1 pattern shares many features with both of the other 2 patterns, it possibly represents a mixture of the other patterns present in polyclonal human serum samples. Further work is now necessary to define the molecular targets of antibodies associated with the identified MOG isoform binding patterns. However, it is a limitation of our study that we were not able to perform these experiments with all samples because of limited availability of samples and reagents.

Our data indicate that these antibody binding patterns are associated with clear structural differences, and therefore, it seems very likely that they represent different antibody clones. The observed MOG isoform binding patterns could be explained by the differential recognition of MOG dimers or multimers caused by clustering of MOG isoforms.^37^ This explanation is supported by a recent study demonstrating that the second hydrophobic domain of MOG (only present in α1 and β1) enhanced the recognition of the extracellular part of MOG by human MOG-IgG.^39^ Moreover, MOG-IgG from most patients may require bivalent binding to MOG dimers, whereas a smaller subset of MOG-IgG shows monovalent binding to monomers such as the 8-18-C5 monoclonal antibody.

To conclude, our novel finding of differential MOG isoform binding patterns could explain previous discrepant reports and instruct future studies to improve and refine MOG-IgG antibody assays.

## Glossary

ADEM: acute disseminated encephalomyelitis
CBA: cell-based assay
FACS: fluorescence-activated cell sorting
HCs: healthy controls
IF: immunofluorescence
MOG: myelin oligodendrocyte glycoprotein
MOGAD: MOG-IgG–associated disorders
MOG-ecIgD: extracellular MOG immunoglobulin domain
MOG-IgG: serum IgG antibodies against MOG
MS: multiple sclerosis
NMOSD: neuromyelitis optica spectrum disorder
ON: optic neuritis
PCA: principal component analysis
